# The effect of Lipoxin A_4 _on the interaction between macrophage and osteoblast: possible role in the treatment of aseptic loosening

**DOI:** 10.1186/1471-2474-10-57

**Published:** 2009-06-02

**Authors:** Gang Li, Ping Wu, Yao Xu, Yan Yu, Li Sun, Liang Zhu, Duyun Ye

**Affiliations:** 1 Department of surgery, Liyuan Hospital, Huazhong University of Science and Technology, Wuhan, 430077, PR China; 2 Health Sciences Center and VA Medical Center, University of Utah, Salt Lake City, 84132, USA; 3 Department of Pathophysiology, Tongji Medical College, Huazhong University of Science and Technology, Wuhan, 430030, PR China; 4 Seven year system of clinical medicine, Tongji Medical College, Huazhong University of Science and Technology, Wuhan, 430030, PR China

## Abstract

**Background:**

Aseptic loosening (AL) is the main problem of total joints replacement (TJR) by the implantation of permanently prosthetic components. In vitro and in vivo studies have clearly demonstrated that wear debris and its byproducts could trigger inflammation in the peri-implant tissue. Lipoxins (LXs) are endogenous eicosanoids synthesized locally from arachidonate acid (AA) at sites of inflammation and mediate pro-resolving activity. A number of studies have demonstrated the effect of LXA_4 _to counteract inflammation in different cell and animal models, but till now, no relative report about the role of LXs in progress or prevention of AL.

**Methods:**

Murine RAW264.7 macrophage cell line and MC3T3-E1 osteoblasts (OB) cell line were purchased. Co-cultured model of these two cell lines was established. To explore the effect of exogenous Lipoxin A_4 _(LXA_4_) on polymethylmethacrylate (PMMA) induced inflammation, pro-inflammatory cytokines including TNF-α, IL-1β, PGE_2 _and GM-CSF were measured by ELISA kits and bone resorption was quantified by measuring calcium release from 5-day-old mice calvaria in vitro. To determine further the endogenous effect of LXA_4_, cells were co-cultured and with or without 15-lipoxygease (15-LO) blocking by 15-LO siRNA. Both real-time PCR and western blotting were applied to confirm the inhibitory efficiency of 15-LO by siRNA.

**Results:**

0.1 mg/ml, 0.5 mg/ml and 1.0 mg/ml PMMA showed a time-dependent manner to trigger production of all the pro-inflammatory cytokines studied. Exogenous 0–100 nM LXA_4 _presented an inhibitory effect on both generation of above cytokines and PMMA stimulated calvarial bone resorption with a dose-dependent manner. LXA_4 _in supernatant from neither rest macrophages nor macrophages cultured alone exposing to PMMA was detectable. In co-cultured cells challenged by PMMA, LXA_4 _was increased significantly, while, this enhance could be partly inhibited by 15-LO siRNA. When LXA_4 _generation was blocked with 15-LO siRNA, the PMMA induced pro-inflammatory cytokines were elevated and bone resorption was accelerated.

**Conclusion:**

In the present study, we demonstrated that LXA_4 _had a favorable inhibitory effect on PMMA-induced inflammation in a macrophage and OB co-culture system.

## Background

The total joints replacement (TJR) by the implantation of permanently prosthetic components has been one of the most successful clinical procedures in orthopaedic surgery of recent decades[1]. However, despite the clinical effectiveness of joint replacement arthroplasty, aseptic loosening (AL) of the prosthesis still remains a major problem, especially for long-term success and survival of prosthesis[2]. In most cases of AL, revision surgery is needed, which will cause serious damage physically and mentally to the sufferers[3]. Among all the reasons of AL, inflammatory reaction induced by excessive production of wear particles from the implant components and consequent peri-implant osteolysis are believed to be the primary causes [4,5].

In vitro and in vivo studies have clearly demonstrated that wear debris and its byproducts could trigger a series of cellular biology responses[6]. Phagocytic cells engulfing particulate debris become activated, release pro-inflammatory cytokines, degradative enzymes, reactive oxygen radicals and other substances, stimulate osteoclasts to undermine the prosthetic bed [7-9]. Of the entire cellular population in the foreign body and chronic inflammation to wear particles, macrophages comprise 60–80% [10-13].

While most of the studies have been concentrated on understanding excessive bone resorption in osteolysis, less attention has been paid to the possible involvement of defective bone formation[14]. As we know, normally, the balance between bone resorption and formation leads to the bone homeostasis[15,16]. It is therefore also critical to consider the effect of wear debris on osteoblasts (OBs), the cell type responsible for bone formation. Interestingly, it has been demonstrated that following the phagocytosis of polymethylmethacrylate (PMMA) by macrophages, OBs are necessary to stimulate osteoclast generation and migration in the peri-implant tissue [17-19]. These prior studies shed some light on the importance of the OBs response to mediators released by macrophages in AL; unfortunately, they do not take into account the potential effects of mediators released by osteoblasts upon macrophages.

Lipoxins (LXs) are endogenous eicosanoids that were synthesized locally from arachidonate acid at sites of inflammation and mediate pro-resolving activity [20]. LXA_4_, LXB_4 _and their enantiomers are the major LXs in mammals so far reported[21]. A number of studies have demonstrated the effect of LXA_4 _to counteract inflammation in different cell and animal models, such as asthma, periodontal disease, atherosclerosis, cystic fibrosis, gastrointestinal disease, acute lung injury and rheumatic diseases[20,22-28]. They are considered as endogenous "stop signals" for inflammation, but till now, no relative report about the role of LXs in progress or prevention of AL.

Thus in this study, we applied the co-culture system to test the crosstalk between macrophage and OB and the potential therapeutic effect of LXs on these cells. The present study was an initial research to examine the inhibitory effect of LXs on AL.

## Methods

### Preparation of PMMA particles and LXA_4_

Spherical PMMA particles (Polysciences, USA), with mean diameter 6.0 ± 1.8 μm, were measured by a Coulter Multisizer II (Coulter Electronics) and it was confirmed that ninety percent of the particles were <10 μm in diameter. The particles were rinsed in 70% ethanol for three times and sterilized in 70% ethanol for 48 h, then washed three times in sterile phosphate buffered saline (PBS). A Limulus Amebocyte Lysate kit (BioWhittaker, USA) was used to test negative for endotoxin. After that, the particles were then suspended in sterile PBS at 5 mg/ml (1 × 10^8 ^particles/ml) till use.

LXA_4 _(Cayman Chemical Company, USA) was stored at -80°C until being diluted in serum-free culture medium immediately before use.

### Cell Culture and Preparation of Conditioned Medium

The RAW264.7 murine macrophage cell line (The Cell Bank of Type Culture Collection of Chinese Academy of Sciences, China) was cultured as we did before[29]. Cells were cultured at 37°C in RPMI-1640 (Gibco BLR Life Technologies Inc, USA) containing 10% heat-inactivated fetal calf serum (FCS) (Sigma Chemical Co, USA), 2 g/L NaHCO_3 _(pH = 7.4), penicillin (100 units/ml) and streptomycin sulfate (100 μg/ml) in a humidified atmosphere of 5% CO_2_.

The MC3T3-E1 murine OB cell line (China Center for Type Culture Collection) was used in this study because it bore many similarities to OB, such as morphology, production of calcified bone matrix, collagen, and alkaline phosphatase [30]. These cells were maintained in DMEM (Gibco BRL, USA) with 10% FCS, OPI Media Supplement (1 mM oxaloacetate, 0.45 mM pyruvate, 0.2 U/ml insulin, Sigma, USA) and antibiotics. Cells were kept in an incubator as described above.

Co-culture model was established as the following[30]. Macrophages were seeded in Falcon 6-well plates (BD Bioscience, USA) at a density of 2 × 10^6 ^cells per well with 3 ml of medium. Simultaneously, OBs were seeded onto 6-well culture dish inserts (BD Bioscience, USA) at a density of 3 × 10^5 ^cells per insert with 2 ml of media. The insert contained a membrane at the bottom onto which cells could grow but was semi-permeable to soluble factors. Macrophages and OBs were incubated separately for 24 hours then rinsed twice with sterile PBS. After that, PMMA particles in DMEM/10% FCS were added to the macrophages. Fresh medium was added to the OBs. The inserts with the OBs were then placed into the culture dishes which contained the macrophages and particles. The co-cultured cells were incubated for additional 24 or 48 hours.

### ELISA

The conditioned medium were collected, filtered through a 0.22 mm filter, and then assayed for some inflammation-related cytokines. LXA_4 _concentration in cell supernatants was determined using ELISA kit (Oxford Biomedical Research, USA). TNF-α, IL-1β, PGE_2 _and GM-CSF were determined using ELISA kit (Pierce, USA). All above tests were accomplished according to the manufacturer's instructions.

### Gene Silencing

Based on the previous report[31], a siRNA against mice 15-lipoxygenase (15-LO) was chemically synthesized and purified (GeneChem, Inc., Shanghai, China). The sequences of the siRNA oligos used were following: 15LO-siRNA-233: sense 5'-GCA ACU GGA UUU CUG UGA AGG-3', antisense 3'-CGU UGA CCU AAA GAC ACU UCC-5'; 15LO-siRNA-826: sense 5'-GAA GCG GAU UUC UUC CUU CUG-3', antisense 3'-CUU CGC CUA AAG AAG GAA GAC-5'; Scramble siRNA: sense 5'-GAU GCG GAA UUG UUC CUA CUG-3', antisense 3'-CUA CGC CUU AAC AAG GAU GAC-5'. The mix of 15LO-siRNA-233 and 15LO-siRNA-826 or negative control of scramble siRNA in serum-free medium was transfected into RAW 264.7 macrophages using Lipofectamine 2000 (Invitrogen, USA). 4 hours after then, medium was changed to complete medium and cells were allowed to recover for additional 24 hours prior to co-cultured with OBs or 15-LO mRNA measuring, or 48 hours prior to 15-LO protein measuring.

### Real time quantitative reverse transcriptase-polymerase chain reaction (RT-QPCR)

Total cellular RNA was isolated from cultured cells using RNeasy mini kit (Qiagen Inc., USA). First strand cDNA was reverse transcribed from 2.0 μg of total RNA using a high capacity cDNA archive kit (Applied Biosystems, USA). MRNA level of 15-LO was quantified with specific primers: sense 5'-ACC CCA CCG CCG ATT TT-3', antisense 5'-AGC TTC GGA CCC AGC ATT T-3'. GAPDH was applied as internal control with specific primers: sense 5'-TGT GTC CGT CGT GGA TCT GA-3', antisense 5'-CCT GCT TCA CCA CCT TCT TGA T-3'[32,33]. CDNA (90 ng) was mixed with ABI TaqMan Universal PCR Master Mix and the appropriate ABI TaqMan Gene Expression Assay for the gene of interest. We used the comparative cycle threshold (C_T_) method (2^-ΔΔCT^) to calculate relative gene expression under experimental and control conditions normalized to GAPDH. The results were expressed as fold-change over control values[34].

### Calvarial bone resorption assay

Bone resorption was quantified by measuring calcium release from 5-day-old mice calvaria in vitro[35]. Briefly, halved calvaria were cultured singly on stainless steel grids in 30-mm dishes with 1.5 ml BGJb medium (Sigma Chemical Co, USA) supplemented with 5% complement-inactivated FCS, 1% penicillin/streptomycin and 50 mg/ml ascorbic acid (Sigma Chemical Co, USA). The plates were then incubated for 24 hours at 37°C with 5% CO_2_. Then the media were aspirated and the wells and disks were rinsed twice with sterile PBS. A 1:1 mixture of different conditioned medium to BGJb/FCS/Antibiotic, and a 1:1 mixture of DMEM/FCS with BGJb/FCS/Antibiotic were placed in control plates. The calvaria were cultured for a further 48 hours and then the calcium content of the media were measured by automated colorimetric assay. In all assays, part of calvaria remained unstimulated to provide a measure of spontaneous calcium release from bone, and part of calvaria were cultured in the presence of 1 mM PGE_2 _to demonstrate that the bone explants were metabolically responsive.

### Western immunoblotting

Cell lysates were prepared in RIPA buffer supplemented with a full spectrum protease inhibitor cocktail (Roche Biochemicals, USA), and then subjected to SDS-PAGE electrophoresis and subsequent membrane transfer. Before probing with Abs, the blotted membranes were blocked with 3% (w/v) BSA in 1% TBS-Tween 20 for 1 h. Mice anti-15-LO antibodies (Cell Signaling Technologies, USA) were used for membrane immunostaining. After washing 3 times with TBS-Tween 20 at room temperature, the membranes were incubated for 1 h with a HRP-conjugated secondary Ab. Membranes were washed three times in TBS-Tween 20 and developed with ECL reagent (Millipore). Appropriate dilutions of antibody were empirically derived.

All above studies were approved by the Ethical Committee of Tongji Medical College, Huazhong University of Science and Technology, China.

### Statistical analysis of the data

Statistical analysis among groups was performed by one-way ANOVA test. Data were expressed as mean ± standard error of the mean. A P-value of less than 0.05 was considered as significant difference.

## Results

### LXA_4 _inhibited the pro-inflammatory cytokines induced by PMMA

**Figure 1:**
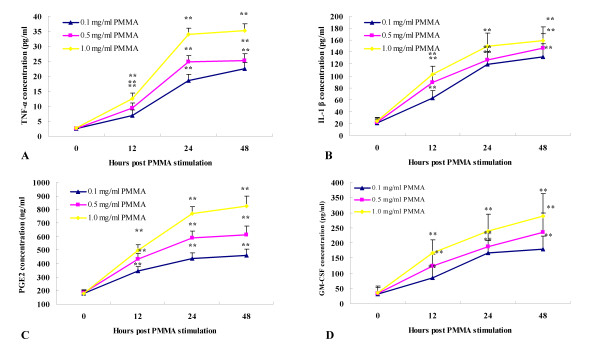
**Changes of pro-inflammatory cytokines in culture media of macrophages exposed to different concentration of PMMA**. All the cytokines were measured with correspondent ELISA kits. **, *P *< 0.001 compared to 0 hour group.

PMMA particles were widely used in TJR and generally found in peri-prosthetic tissue. Thus in our study, they served as stimulator to trigger the inflammation of macrophages. Different concentration of PMMA (final concentration: 0.1 mg/ml, 0.5 mg/ml and 1.0 mg/ml) were added into RAW 264.7 culture medium 12, 24 or 48 hours prior to test. It was confirmed that (Fig. 1), in all of the PMMA concentration groups, the pro-inflammatory cytokines were increased in a time-dependent manner in 12 hours to 48 hours after treatment (*P *< 0.001 compared to control groups). The effect of PMMA at a concentration higher than 1 mg/ml was also studied. It was confirmed that 1 mg/ml PMMA did not reach the highest plateau (data not shown).

**Figure 2:**
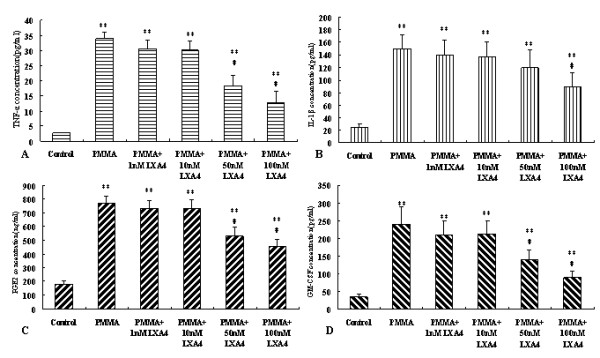
**LXA_4 _inhibited the production of pro-inflammatory cytokines in culture media of macrophages stimulated with PMMA**. Cells were treated with 1.0 mg/ml PMMA and different dose of LXA_4 _for 24 hours. **, *P *< 0.001 compared to control group; #, P < 0.05 compared to cells treated with PMMA only.

Then, in order to study the effect of LXA_4_, 1.0 mg/ml PMMA was applied to stimulate the macrophages, while, 0–100 nM LXA_4 _was administrated simultaneously. After 24 hours, as seen in Fig. 2, LXA_4 _showed inhibitory effect on 1.0 mg/ml PMMA-induced pro-inflammatory cytokines production in a dose-dependent manner. Compared with the cells without LXA_4 _treatment, TNF-α, PGE_2 _and GM-CSF in 50 nM LXA_4 _treated cells and TNF-α, IL-1β, PGE_2 _and GM-CSF in 100 nM LXA_4 _treated cells were significantly lower (*P *< 0.05).

### LXA_4 _blocked the bone resorption induced by PMMA

**Figure 3:**
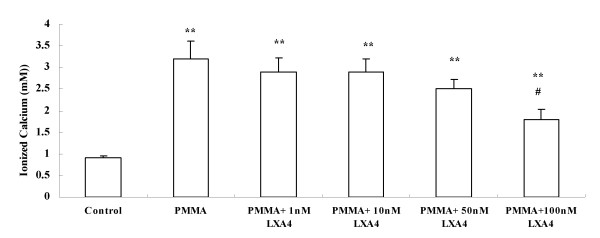
**LXA_4 _blocked PMMA stimulated calvarial bone resorption**. Each point represented the mean ± standard error of mean of 5-calvarial halves. **, *P *< 0.001 compared to control group; #, *P *< 0.05 compared to cells treated with PMMA only.

Since the resorptive activity of culture media from macrophages exposed to diverse types of particles had been documented[36], we further studied the effect of LXA_4 _on in vitro calvarial bone resorption. The culture media from control cells, PMMA stimulated cells or both PMMA and LXA_4 _treated cells were added into calvarial bone tissue, respectively. It was shown in Fig. 3, after 48 hours treatment, ionized calcium level was obviously increased from 0.91 ± 0.05 mM in control groups to 3.22 ± 0.42 mM in 1.0 mg/ml PMMA stimulated groups (*P *< 0.01), while, LXA_4 _in all concentrations decreased the calcium enhance. Statistical difference could be seen in 100 nM LXA_4 _treated group.

### Effect of PMMA on endogenous LXA_4 _production

**Figure 4:**
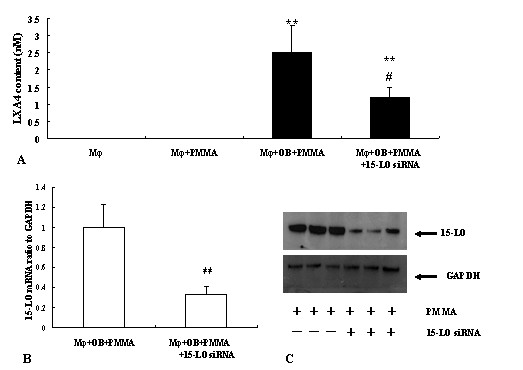
**Effect of PMMA on endogenous LXA_4 _production**. A, LXA_4 _content in culture media was monitored with ELISA kit. **, *P *< 0.01 compared to macrophages+PMMA; #, *P *< 0.05 compared with macrophage+OB+PMMA. B, Inhibition of 15-LO siRNA on 15-LO mRNA expression measured by RT-QPCR. Results were normalized to GAPDH and expressed as fold induction over cells co-cultured with OB without 15-LO siRNA. C, Inhibition of 15-LO siRNA on 15-LO protein expression measured by western blotting. GAPDH was applied as internal control.

Above data showed an important anti-inflammatory role of exogenous LXA_4 _on the PMMA induced inflammation in cultured macrophages. To determine whether endogenous LXA_4 _formation also played a role in the resolution of peri-implant inflammation induced by wear debris, we next determined weather PMMA could change the production of LXA_4 _in RAW 264.7 cells. RAW 264.7 macrophages were cultured alone or co-cultured with MC3T3-E1 OB cell line and treated with 1.0 mg/ml PMMA for 48 hours. It was found that, in supernatant from macrophages cultured alone, LXA_4 _could be detected in neither control cells nor cells treated with PMMA (Fig. 4.). In supernatant from co-cultured cells exposed to PMMA, LXA_4 _was increased significantly, while, this enhance could be partly inhibited by 15-LO siRNA, which could block the expression of 15-LO, a key enzyme to LXs production in macrophages[31]. The block of 15-LO expression by 15-LO siRNA in macrophages were confirmed by RT-QPCR and western blotting (Fig. 4B and 4C).

### Effect of endogenous LXA_4 _on PMMA induced inflammation in macrophages

**Figure 5:**
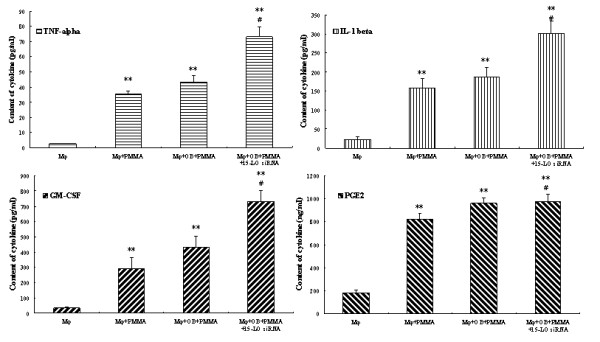
**Effect of endogenous LXA_4 _on the production of pro-inflammatory cytokines**. Culture media were collected 24 hours after treatment. **, *P *< 0.01 compared to control Mφ cells; #, *P *< 0.05 compared to Mφ+OB+PMMA cells.

Now that we found LXA_4 _could be generated in macrophages exposed to both OB and PMMA, we further tested the effect of endogenous LXA_4 _on PMMA induced inflammation. Macrophages were co-cultured with OB and treated with 1.0 mg/ml PMMA with or without 15-LO siRNA 24 hours prior to culture media being collected. As seen in Fig. 5, except PGE_2_, all other pro-inflammatory cytokines studied were obviously higher when 15-LO was blocked.

### Effect of endogenous LXA_4 _on PMMA induced bone resorption

**Figure 6:**
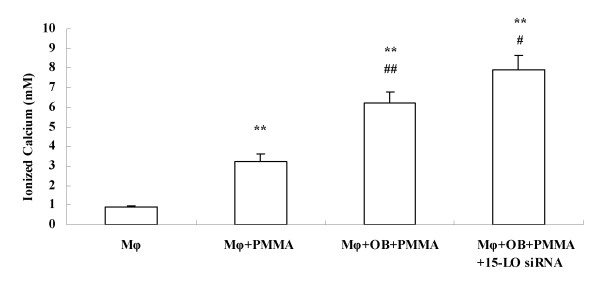
**Effect of endogenous LXA_4 _on PMMA induced bone resorption**. **, *P *< 0.01 compared to Mφ group; #, *P *< 0.05 compared to Mφ+OB+PMMA group; ##, *P *< 0.05 compared to Mφ+PMMA group.

Because increase of pro-inflammatory cytokines paralleled bone resorption in both AL patients and our above results[30], we next examined the contribution of endogenous LXA_4 _to PMMA induced bone resorption. The data showed that 15-LO siRNA significantly accelerated the bone resorption (Fig. 6, *P *< 0.05). Very interesting, we also found that after PMMA stimulation, media from co-cultured cells lead to obviously higher calcium content than media from macrophages cultured alone (Fig. 6, *P *< 0.05).

## Discussion

Substantial progress has been made in recent years elucidating the mechanisms responsible for AL. One area has expanded rapidly is the demonstration that pro-inflammatory cytokines are not only produced in response to wear particles but also responsible for the downstream processes leading to osteolysis [37,38]. Undoubtedly, anti-inflammation is a good therapeutic option deserving attention. Some of the anti-inflammatory agents have yielded encouraging results in animal models of AL. For example, Etanercept[39] and pentoxifylline[40], a decoy receptor for TNF and an inhibitor of TNF secretion, respectively, have been independently shown to attenuate particle-induced osteolysis in the murine calvarial model, along with COX2 inhibitor Celecoxib[41].

However, despite these encouraging animal studies suggesting an association between AL and pro-inflammatory cytokines, no approved treatments are proved to perform in the prevention or treatment of human osteolysis[14]. Orally administration of pentoxifylline can reduce wear debris induced inflammation in isolated monocytes from healthy subjects[42]. But we do not know weather it could also work in patients with AL. A small clinical trial about Etanercept in osteolysis patients proved inconclusive[43]. Although it is found that systemic trafficking of macrophages to peri-implant tissue could be induced by bone cement particles in mice[12], there is no clear evidence in favor of systemic elevated levels of pro-inflammatory markers in serum of patients[14,44-46]. Meanwhile, even if multiple pro-inflammatory cytokines, such as IL-1, IL-6 and TNF-α, are crucial to osteolysis and ultimately AL, the synergistic interactions among these cytokines are required. Knocking out of any one of these cytokines or their receptors dose not completely protect murine calvaria from particle-induced osteolysis[38]. For example, in the murine femoral model, knock out of the IL-1 receptor blocked particle-induced inflammation but not osteolysis[47]. Similarly, neutralizing antibodies to IL-1 did not block osteolysis in an organ culture model of aseptic loosening[48]. Even in the experiments in double knock out mice lacking both IL-1 receptor and IL-6 or both TNF receptor-1 and TNF receptor-2 osteolysis also was not substantially altered[38].

Current unsatisfactory situation of AL prevention and treatment urges us to go back and ask help from the progress in study on mechanism of inflammation itself. As we know, a well-integrated host inflammatory response is essential in maintaining health and fighting disease[49]. Acute inflammation has several outcomes that include progression to chronic inflammation, scarring and fibrosis or complete resolution[50]. Resolution by precise definition and characterization is not the same as endogenous anti-inflammation. It is defined as a highly coordinated and process involving changes from gene transcription to local mediators generation within the resolution phase, not just in vivo dwindling with time of chemotaxic stimuli at the site of inflammation [50-52]. Importantly, successful resolution will limit excessive tissue injury and give little opportunity for the development of chronic, immune-mediated inflammation, like in AL[53].

Based upon previous understanding on inflammation, many drugs try to tame inflammation by inhibiting what occurring at the beginning of the immune response, such as blocking TNF-α or IL-1[38]. However, rather than nip an inflammation in the bud – which may thwart the body's own attempt to heal – a better approach may be to enhance the activity of these natural resolution-promoting compounds. Hence, resolution-directed therapeutics have become a new but attractive terrain for drug design in inflammation related diseases[53,54]. As important lipid pro-resolution mediators, LXs are found to accelerate resolution in different cell and animal models, such as asthma, periodontal disease, atherosclerosis, cystic fibrosis, gastrointestinal disease, acute lung injury and rheumatic diseases[20,22-28]. But till now, no similar study on AL was reported.

Then, after it was confirmed that, in our cell model, PMMA showed a time-dependent manner to trigger production of all the pro-inflammatory cytokines studied, we administrated exogenous LXA_4 _to make sure if it could inhibit PMMA induced inflammation in cultured macrophages. As shown in the results part, LXA_4 _presented an inhibitory effect on both generation of above cytokines and PMMA stimulated calvarial bone resorption with a dose-dependent manner.

Since LXs are important endogenous lipids generated by 5- and 15-LO[55,56], we explore further the role of PMMA on LXA_4 _production in macrophages. It was found that LXA_4 _in culture media from neither rest macrophages nor macrophages cultured alone exposing to PMMA was detectable. Macrophages could only secrete LXA_4 _after exposed to PMMA when they were co-cultured with OBs. This might because LXs are transcellular metabolism of AA by LO/LO interaction pathways in different cells[53]. Next, we also found that when LXA_4 _generation was blocked with 15-LO siRNA, which could down-regulate both mRNA and protein expression in macrophages as shown in Fig. 4, the PMMA induced inflammation became more serious. The changes included elevated pro-inflammatory cytokines and accelerated bone resorption.

It is clear that exogenous LXA_4 _at the concentration of 1 to 10 nM did not show any obvious influence on PMMA-induced cytokine production or bone resorption (Fig. 2 and 3), while, the concentration of LXA_4 _produced by co-culture system exposed to PMMA was just 2.5 ± 0.8 nM (Fig. 4). This concentration difference may be because that LXA_4 _is a short-acting lipid, both *in vitr*o and *in vivo*[57]. Exogenous LXA_4 _could be rapidly converted by initial dehydrogenation at carbon 15 to 15-oxo-LXA4 which is biologically inactive [58], but endogenous LXA_4 _was produced persistently.

We also found another interesting phenomena that when macrophage were co-cultured with OBs, after PMMA challenge, both pro-inflammatory cytokines and bone resorption level are higher than which are in macrophages alone. Combining with what we concluded above that the cooperation of OBs to macrophages was also necessary to generation and effect of LXA_4_, it indicated that the micro-circumstance in the peri-implant tissue contribute not only to the beginning of particles-induced inflammation but also the resolution after that. The balance between these two parts decides the patients' destiny after TJR. It also showed that peri-implant inflammation could be better reproduced by this co-culture model.

Finally, it should be pointed out that in this paper we just used PMMA particles to mimic the inflammation in macrophages and OBs which participate in periprosthetic inflammation of AL patients. A related limitation is that it could not present all the changes in patients who use prosthesis made of other materials. Some other products of 15-LO, like 15-HETE and hepoxilins[59], may also contribute to the PMMA-induced inflammation in the co-culture system we used here. The underlying mechanisms may vary in different prosthesis components, even in different size and dose of same particles[30,60]. And, differences between the in vivo and the cell culture study methods make it still too early to conclude whether LXs contribute to prevention or treatment of AL.

## Conclusion

Taken together, our findings indicate that LXA_4 _has an inhibitory effect on PMMA-induced inflammation in a macrophage and OB co-culture system. Although it is now hard to say that LXA_4 _could be used in the treatment of patient suffered from AL, this study is a valuable attempt in searching for alternative therapeutic strategy involving endogenous anti-inflammatory and pro-resolving lipid mediators.

## Competing interests

The authors declare that they have no competing interests.

## Authors' contributions

PW conceived of the study and participated in its design and helped to draft the manuscript. GL carried out the ELISA assay. YX carried out cell culture. YY carried out establishing the co-culture model. LS carried out real-time PCR. LZ carried out western-blotting. DY carries out the design of the study. All authors have read and approved the final manuscript.

## Pre-publication history

The pre-publication history for this paper can be accessed here:


